# Comparison of antibiotic resistance patterns in collections of *Escherichia coli* and *Proteus mirabilis* uropathogenic strains

**DOI:** 10.1007/s11033-012-2420-3

**Published:** 2013-01-04

**Authors:** Wioletta Adamus-Bialek, Elzbieta Zajac, Pawel Parniewski, Wieslaw Kaca

**Affiliations:** 1 Department of Environment Protection and Modelling, Jan Kochanowski University, 15 Swietokrzyska Street, 25-406 Kielce, Poland; 2 Institute of Mathematics, Jan Kochanowski University, 15 Swietokrzyska Street, 25-406 Kielce, Poland; 3 Laboratory of Molecular Genetics, Institute of Medical Biology, Polish Academy of Sciences, Lodz, 106 Lodowa Street, 93-232 Lodz, Poland; 4 Department of Microbiology, Jan Kochanowski University, 15 Swietokrzyska Street, 25-406 Kielce, Poland

**Keywords:** *Proteus mirabilis*, *Escherichia coli*, UTI, Antibiotic resistance, Association analysis

## Abstract

*Escherichia coli* and *Proteus mirabilis* are important urinary tract pathogens. The constant increase in the antibiotic resistance of clinical bacterial strains has become an important clinical problem. The aim of this study was to compare the antibiotic resistance of 141 clinical (Sweden and Poland) and 42 laboratory (Czech Republic) *P.* *mirabilis* strains and 129 clinical (Poland) uropathogenic *E. coli* strains. The proportion of unique versus diverse patterns in Swedish clinical and laboratory *P. mirabilis* strain collections was comparable. Notably, a similar proportion of unique versus diverse patterns was observed in Polish clinical *P. mirabilis* and *E. coli* strain collections. Mathematical models of the antibiotic resistance of *E. coli* and *P. mirabilis* strains based on Kohonen networks and association analysis are presented. In contrast to the three clinical strain collections, which revealed complex associations with the antibiotics tested, laboratory *P. mirabilis* strains provided simple antibiotic association diagrams. The monitoring of antibiotic resistance patterns of clinical *E. coli* and *P. mirabilis* strains plays an important role in the treatment procedures for urinary tract infections and is important in the context of the spreading drug resistance in uropathogenic strain populations. The adaptability and flexibility of the genomes of *E. coli* and *P.* *mirabilis* strains are discussed.

## Introduction

Human urinary tract infections (UTIs) are among the most common bacterial diseases [1, 2]. Persistent recurrences and asymptomatic infections are responsible for the difficult treatment of UTIs. This is mostly due to the presence of uropathogenic *Escherichia coli* and *Proteus mirabilis* strains in the urinary tract. *E. coli* accounts for 80 % of all bacteria isolated from the urine. *P. mirabilis* strains cause 10 % of uncomplicated urinary tract infections [3]. They are the fifth most common cause of nosocomial urinary tract infections and sepsis in hospitalized individuals [4, 5]. *E. coli* strains represent many different intestinal and extraintestinal pathotypes (IPEC and ExPEC, respectively) that are responsible for numerous diseases. Uropathogenic *E. coli* strains (UPECs) are ExPEC and constitute the most distinct phylogenetic pathotype among *E. coli* [6]. Importantly, uropathogenic *P. mirabilis* and *E. coli* strains may also manifest resistance to several antimicrobial agents, including extended-spectrum cephalosporins, fluoroquinolones, and aminoglycosides [7–9]. The increase in drug resistance has become a serious problem in effective antibiotic administration [10].

Although the molecular mechanisms of bacterial antibiotic resistance are known, they remain the object of studies worldwide. Several mechanisms explaining the emergence of drug resistance have been discovered in the past decades, the best known being beta-lactamase and quinolone resistance mechanisms. The presence of beta-lactamases and complex “efflux pumps” is considered to be the mechanism of beta-lactam resistance [7]. Beta-lactamases are enzymes that break the beta-lactam ring and deactivate this class of antimicrobial drugs [11]. Beta-lactamases are divided into four molecular classes (A, B, C, and D). Their molecular homology is diverse, and they do not seem to have one common ancestor. The presence of a serine residue in the active center is typical of A, C, and D classes, while in class B beta-lactamases, zinc ions are required. Beta-lactamases are encoded by either chromosomes or plasmids. The highly mobile nature of beta-lactamase genes remains an important problem in UTI treatment [8, 10].

The complex characteristics of bacterial antibiotic resistance may be analyzed by mathematical methods to model the dynamics of this process and anticipate its development [12, 13]. There is a need to create a tool for the development of a strategy against the spread of multi-drug-resistant (MDR) strains. In this work, the Kohonen network method was used to identify similar groups of antibiotics that were reactive against the investigated strains. To formulate a hypothesis about the dynamics of the resistance patterns, an association analysis of chosen antibiotics was performed for the investigated collections of strains [14].

The drug resistance profiles of *E. coli* strains and *P. mirabilis* strains were analyzed. One of the tasks was to compare the antibiotic resistance patterns of *P. mirabilis* and *E. coli* strains isolated from UTI patients with those of *P. mirabilis* strain collections stored for many years in the laboratory. Finally, a mathematical analysis of the antibiotic resistance patterns of *P. mirabilis* and *E. coli* strains was carried out to identify their correlation with virulence profiles.

## Materials and methods

### Bacterial strain collections

In this study, the following bacterial strains of *E. coli* and *P. mirabilis* were used:

1. A collection of 129 clinical *E. coli* strains isolated from the urine of patients in different wards of Military Teaching Hospital No. 2, Medical University of Lodz, Poland, in 2005–2007.
2. A collection of 43 clinical *P. mirabilis* strains isolated from the urine of patients of the Swietokrzyskie Oncology Center in Kielce, Poland, in 2002.
3. A collection of 99 clinical isolates of *P. mirabilis* strains collected at the Department of Clinical Microbiology of the Karolinska Hospital in Stockholm, Sweden, between October 1999 and January 2000. These strains were isolated from UTI patients and sub-cultured four times prior to the study.
4. A collection of 42 laboratory *P. mirabilis* strains from the Czech National Collection of Type Cultures from the Institute of Epidemiology and Microbiology, Prague, Czech Republic. These strains were stored for more than 20 years and sub-cultured at least 20 times.

For everyday work, the clinical and laboratory strains were stored at 4 °C in bacterial media, and for longer storage, they were stored in glycerol stock solutions at −70 °C.

### Bacterial identification and cultivation media

*Escherichia coli* and *P. mirabilis* strains were identified based on their differential growth on CPS3 medium (bioMerieux). UTI cases were confirmed by the presence of >10^4^ cfu/ml of bacteria in a urine sample. All of the strains were grown at 37 °C on LB plates for further tests.

### Antibiotic susceptibility testing

*Proteus mirabilis* strains were subjected to an in vitro antimicrobial susceptibility disc diffusion test according to the guidelines of the National Committee for Clinical Laboratory Standards [15]. A standardized inoculum of bacteria (0.5 McFarland standard, 1.5 × 10^8^ cfu/ml) was swabbed onto the surface of Mueller–Hinton agar (MHA) plates (Difco Laboratories, Detroit MI, USA). Filter paper discs impregnated with antimicrobial agents were placed on the agar surface. After 16–18 h of incubation at 35 °C, the diameter of the inhibition zone around each disc was measured, and these measurements were compared with the NCCLS disc diffusion tables [15].

The drug resistance of *E. coli* strains was determined for enterobacteria of urinary origin by a susceptibility test (ATB UR5, bioMerieux) at the Faculty of Laboratory Diagnostics and Clinical Biochemistry, Military Teaching Hospital No. 2, Medical University of Lodz, Poland.

Bacterial isolates were determined to be sensitive (S), moderately sensitive (M), or resistant (R) to the antimicrobial agents tested.

### Antimicrobial agents

Antimicrobial discs with ampicillin (AP) 10 μg, cotrimoxazole (trimethoprim/sulfamethoxazole) (TS) 1.25/27.75 μg, nitrofurantoin (NI) 30 μg, norfloxacin 10 μg, carbenicillin (PY) 100 μg, ofloxacin (OFX) 5 μg, tetracycline (T) 30 μg, amoxicillin/clavulanate (AUG) 20/10 μg, ciprofloxacin (CIP) 5 μg, amikacin (AK) 30 μg, aztreonam (ATM) 30 μg, cefuroxime (CXM) 30 μg, imipenem (IMI) 10 μg, polymyxin B (PB) 300 μg, and colistin sulfate (CO) 100 μg (Mast Diagnostics, Mast Group Ltd., Merseyside, UK) were used on the studied collections of *P. mirabilis* strains.

*Escherichia coli* strains was screened for their susceptibility to amoxicillin (A), AUG, piperacillin (PIP), cefalotin (CF), cefoxitin (CFX), cefotaxime (CFT), ceftazidime (CFZ), IMI, tobramycin (TB), AK, gentamicin (Gm), netilmicin (NT), nalidixic acid (Na), NOR, CIP, NI, TS and fosfomycin (F). The list of antibiotics used in the study is presented in Table 1.

**Table 1:** List of antibiotics used in the study

Antibiotics used against		Antibiotics by class
*E. coli* strains	*P. mirabilis* strains	
		I. β-lactam antibiotics
Amoxicillin (A)	Carbenicillin (PY)	Penicillins
Piperacillin (PIP)	Ampicillin (AP)	
Amoxicillin/Clavulanate (AUG)		
Cefalotin (1st G) (CF)		Cephalosporins
Cefoxitin (2nd G) (CFX)	Cefuroxime (2nd G) (CXM)	
Cefotaxime (3rd G) (CFT)		
Ceftazidime (3rd G) (CFZ)		
Imipenem (IMI)		Carbapenems
	Aztreonam (ATM)	Monobactams
Amikacin (AK)		II. Aminoglycosides
Tobramycin (TB)		
Gentamicin (Gm)		
Netilmicin (NT)		
Nalidixic acid (Na)		III. Quinolones
Norfloxacin (NOR)		
Ciprofloxacin (CIP)		
	Ofloxacin (OFX)	
	Tetracycline (T)	IV. Tetracyclines
	Polymyxin B (PB)	V. Polypeptides
	Colistin (Polymyxin E) (CO)	
Nitrofurantoin (NI)		VI. Nitrofurans
Cotrimoxazole (TS)		VII. Sulfonamides
Fosfomycin (F)		VIII. Folic acid derivatives

### Mathematical and statistical analysis

The following methods were used in mathematical and statistical analysis: Kohonen networks and association analysis using SAS^®^ Data Miner tools.

## Results

### Antibiotic resistance of clinical and laboratory collections of *P. mirabilis* strains

A comparative analysis (Table 2) showed that two clinical *P. mirabilis* strain collections from Sweden and Poland had much more diverse patterns of resistance than the third collection (laboratory). The number of diverse resistance patterns was as follows: 66 % of all strains (44) in the Polish collection, 36 % of all strains (99) in the Swedish collection and 21 % of strains (42) in the laboratory collection. Among the Polish collection strains, there were 23 unique patterns of resistance, representing 79 % of all patterns of resistance (29). In the Swedish collection, 15 unique patterns of drug resistance were identified, representing 42 % of all patterns of resistance (36). In the laboratory *P. mirabilis* collection, six unique patterns of drug resistance were identified, accounting for 67 % of all patterns of resistance (9) (Table 2).

**Table 2:** Comparison of antibiotic resistance patterns of *E. coli* and *P*. *mirabilis* collections

Strain collections	Antimicrobial resistance patterns	
	No. of unique patterns	No. of diverse patterns
*P. mirabilis*	15	36
99 Swedish clinical strains		
*P. mirabilis*	6	9
42 Czech laboratory strains		
*P. mirabilis*	23	29
44 Polish clinical strains		
*E. coli* 129 Polish clinical strains	63	76

The bacterial strains revealed a high resistance to some of the applied antibiotics (Table 3). Over 80 % of all *P. mirabilis* strains were resistant to T, NI, and polypeptides. The majority of bacterial strains in the Polish collection were resistant to beta-lactam antibiotics: 60 % of Polish *P. mirabilis* strains were resistant to CXM, and almost 50 % of Polish strains were resistant to PY, AP, IMI, and ATM. No single antibiotic was found to be an effective bactericidal agent against all *P. mirabilis* strains in this collection. In the other two collections (laboratory and Swedish), the strains displayed a much higher sensitivity. IMI, ATM and AK were found to be effective bactericidal agents against all *P. mirabilis* strains in these collections. Additionally, in the laboratory *P. mirabilis* collection, all strains were susceptible to AUG, PY, norfloxacin, CIP, OFX and cotrimoxazole.

**Table 3:** Number (percentage) of bacterial strains resistant to the antibiotics used

Antibiotics	No. (%) of resistant strains			
	*P. mirabilis* collections			*E. coli*
	Laboratory	Swedish	Polish	
AUG	0	2 (2.0)	5 (12.0)	8 (6.0)
A	–	–	–	**73 (57.0)**
PIP	–	–	–	27 (21.0)
PY	0	13 (13.0)	19 (44.0)	–
AP	2 (9.0)	15 (15.0)	**21 (48.0)**	–
CF	–	–	–	38 (30.0)
CFX	–	–	–	12 (9.0)
CXM	2 (9.0)	3 (3.0)	**26 (60.0)**	–
CFT	–	–	–	7 (5.5)
CFZ	–	–	–	8 (6.0)
IMI	0	0	**20 (46.0)**	0
ATM	0	0	**21 (49.0)**	–
AK	0	0	10 (23.0)	1 (0.7)
TB	–	–	–	12 (9.0)
Gm	–	–	–	20 (16.0)
NT	–	–	–	4 (3.0)
Na	–	–	–	**65 (51.0)**
NOR	0	3 (3.0)	14 (32.0)	38 (30.0)
CIP	0	3 (3.0)	12 (28.0)	38 (30.0)
OFX	0	2 (2.0)	18 (42.0)	–
T	**38 (90.0)**	**94 (94.0)**	**42 (98.0)**	–
PB	**42 (100)**	**97 (97.0)**	**36 (84.0)**	–
CO	**42 (100)**	**99 (100)**	**39 (91.0)**	–
NI	**42 (100)**	**91 (91.0)**	**39 (91.0)**	24 (19.0)
TS	0	19 (19.0)	18 (42.0)	42 (33.0)
F	–	–	–	2 (1.0)

Antibiotics: *AUG* amoxicillin/clavulanate, *A* amoxicillin, *PIP* piperacillin, *PY* carbenicillin, *AP* ampicillin, *CF* cefalotin, *CFX* cefoxitin, *CXM* cefuroxime, *CFT* cefotaxime, *CFZ* ceftazidime, *IMI* imipenem, *ATM* aztreonam, *AK* amikacin, *TB* tobramycin, *Gm* gentamicin, *NT* netilmicin, *Na* nalidixic acid, *NOR* norfloxacin, *CIP* ciprofloxacin, *OFX* ofloxacin, *T* tetracycline, *PB* polymyxin B, *CO* colistin sulfate, *NI* nitrofurantoin, *TS* cotrimoxazole, *F* fosfomycin; *bold* over 45 % of resistant strains;- not studied

**Fig. 1:**
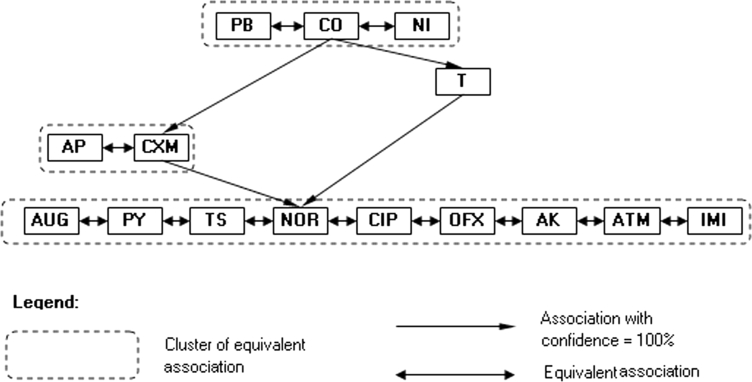
Kohonen map of drug resistance patterns of the *P. mirabilis* laboratory strain collection

**Fig. 2:**
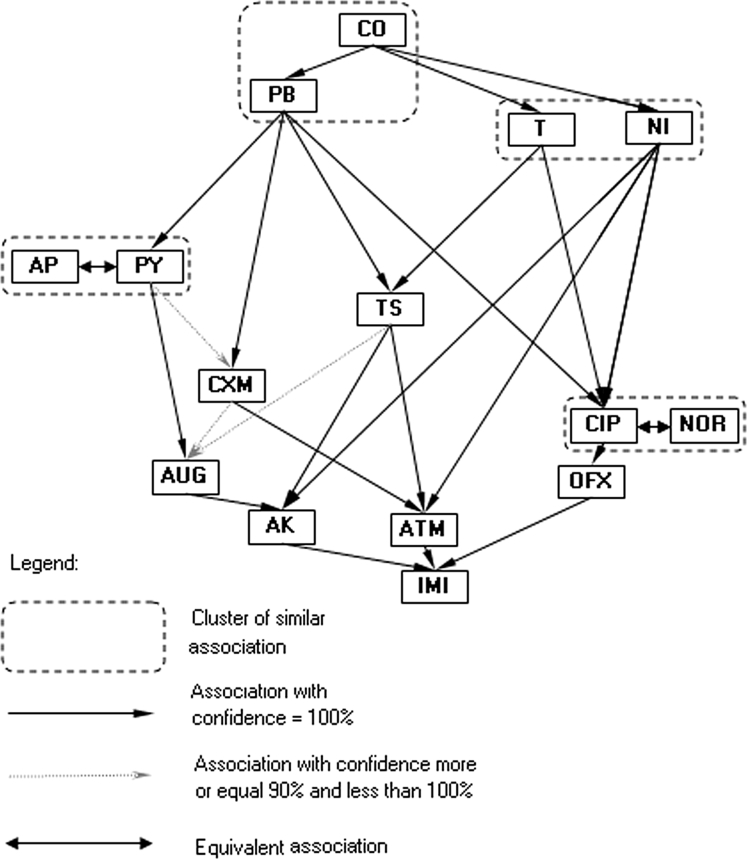
Kohonen map of drug resistance patterns of the *P. mirabilis* swedish strain collection

**Fig. 3:**
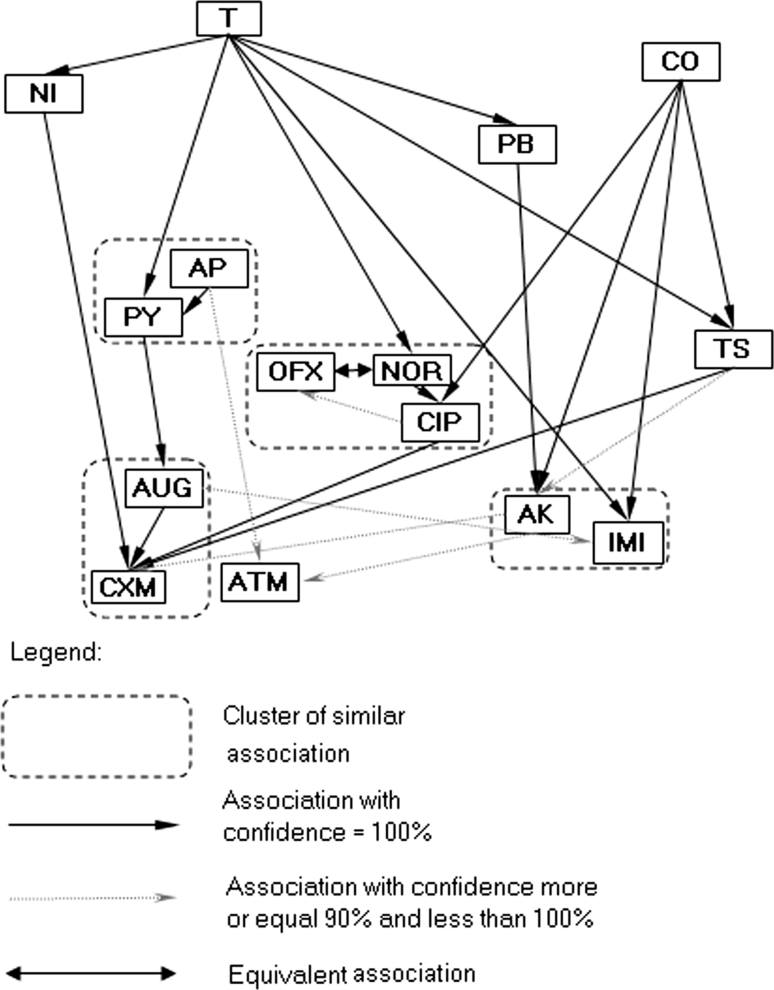
Kohonen map of drug resistance patterns of the *P. mirabilis* polish strain collection

The Kohonen network method and association analysis based on the antibiotic resistance patterns of laboratory *P. mirabilis* strains resulted in a simple association diagram of antibiotic reaction with only three major similarity clusters (Fig. 1). The arrow (e.g. A → B) in the association diagram should be interpreted as follows: “if a strain is sensitive to antibiotic A, then it is sensitive to B” or “if a strain is not sensitive to antibiotic B, then it is not sensitive to A”. For example, resistance to one of the antibiotics from the first cluster (CO; NT or T) was accompanied by a lack of sensitivity to CXM, AP, and T. The analysis did not indicate diverse patterns of antibiotic resistance. This result is in contrast to two the clinical collections, in which much more diverse correlation patterns were observed (Figs. 2, 3). However, similar associations of CO, T, and NOR were observed in the Swedish laboratory collection (Fig. 2). The cluster patterns and association diagrams of the two clinical strain collections differed significantly (compare Figs. 2, 3). Interestingly, antibiotics with similar chemical structures formed one cluster. The diagrams of associations made it possible to present the correlation between antibiotic action patterns and might help to identify hidden relationships in antibiotic resistance mechanisms.

### Antibiotic resistance of the clinical collection of *E. coli* strains

The results demonstrated significantly diverse drug resistance patterns among 129 *E. coli* strains. Sixty-three unique resistance patterns were found, which consisted of 83 % of all diverse drug resistance profiles (76) in these *E. coli* strains. Diverse resistance patterns were found for 59 % of all *E. coli* strains (Table 2).

This collection of strains revealed simultaneous resistance to some of the applied antibiotics (Table 3). *E. coli* strains that were resistant to penicillins were also resistant to quinolones, which was in contrast to cephalosporins and aminoglycosides, which were effective. All of the strains were also susceptible to IMI and AK. More than 70 % of bacterial strains were susceptible to many of the applied antibiotics, with the exception of A (57 % resistant strains), Na (51 % resistant strains), TS (33 % resistant strains), and CF (30 % resistant strains). A total of 60 % of bacterial strains were resistant to at least one beta-lactam antibiotic (A). Moreover, 15 % of the strains (20 isolates) were resistant to at least one aminoglycoside. All bacterial isolates that were resistant to TB were also resistant to Gm. Resistance to quinolones was revealed in 50 % of the strains, and 80 % of these strains were also resistant to A. In conclusion, as many as 50 % of the studied *E. coli* strains displayed an MDR phenotype due to their resistance to at least two antibiotics of two different classes.

**Fig. 4:**
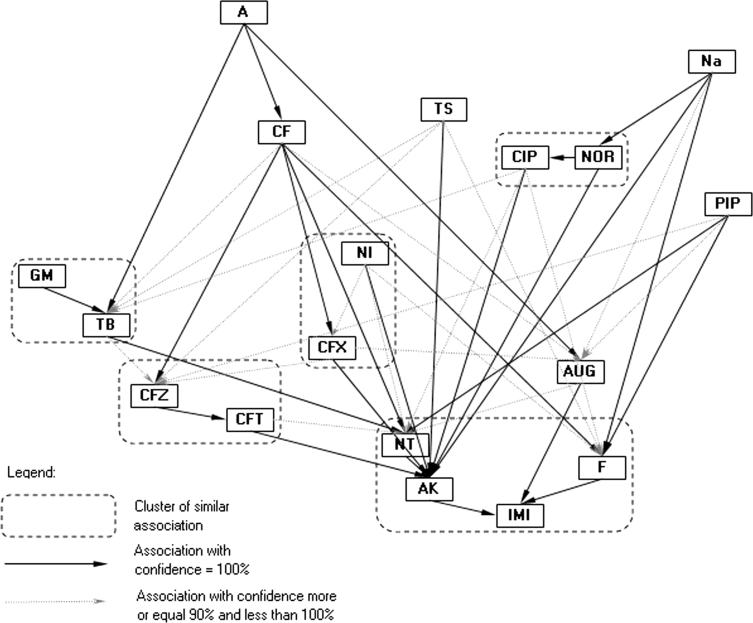
Kohonen map of drug resistance patterns of the *E. coli* polish strain collection

Similar to *P. mirabilis* clinical strains, *E. coli* strains presented a complex pattern of resistance/susceptibility associations (Fig. 4). The mathematical analysis of the resistance of *E. coli* strains showed a much more diverse correlation pattern in contrast to *P. mirabilis* strains. Resistance to A was accompanied by a lack of sensitivity to TB, CF, and AUG. Additionally, resistance to Na was accompanied by a lack of sensitivity to NOR, CIP, AK, fosfomycin (F) and AUG. A weaker correlation was observed for the other antibiotics (TS and PIP). Antibiotics with a similar degree of correlation formed one association cluster. Interestingly, this model indicated that the antibiotics NT, AK, IMI and F rarely led to resistant strains. In contrast, other antibiotics, including A, Na and TS, frequently induced resistance. This finding is consistent with the data shown in Table 3. There were substantial differences in resistance associations among four *P. mirabilis* and *E. coli* strain collections with respect to clustering patterns.

## Discussion

*Escherichia coli* and *P. mirabilis* are the most important etiological factors of UTIs. The pathogenicity of these bacteria is specific to uropathogenic strains due to the presence of virulence factors, such as fimbrial adhesins (S and P in UPEC and MR/P and PMF in *P. mirabilis*) [3, 6]. Toxins, such as α-hemolysin, cnf1, and bacteriocin *usp*, are some of the typical pathogenic factors of *E. coli*, while urease, protease, and hemolysins are characteristic of *P. mirabilis*. It has been shown that two major groups of *E. coli* strains can invade human urinary tracts. The first group is characterized by a statistically limited presence of virulence factors and a multi-drug resistance pattern (including resistance to quinolones). The second *E. coli* group encodes many virulence factors but is susceptible to quinolones and many other antibiotics [16–18]. This finding may imply that the latter group consists of uropathogenic *E. coli.*

A comparison of the antibiotic resistance patterns of one *E. coli* and three *P. mirabilis* collections (Table 2) revealed that the percentage of unique versus diverse patterns in the Swedish clinical and laboratory *P. mirabilis* strain collections remained at the same level. Interestingly, a similar proportion was observed for Polish clinical *E. coli* and *P. mirabilis* collections. This diversity may result from the types of antibiotics and the frequency of their use in Poland and Sweden. In addition, it was shown that there are some groups of antibiotics to which bacterial strains were rarely resistant. If a strain was resistant to an antibiotic in the group, that strain was usually resistant to the majority of antibiotics within the same group (see Figs. 1, 2, 3, 4). The association graphs demonstrated a high probability of co-existing resistance toward antibiotics in particular strain collections. Resistance often concerned antibiotics with different chemical groups. This finding may be a manifestation of general mechanisms for the acquisition of resistance. Therefore, this association may show some new tendencies of the emergence of drug resistance. Observing the structure of the graphs, there were similarities in associations depending on antibiotic types used.

MDR *Proteus* and *Escherichia* strains pose a serious hazard for patients hospitalized as a result of UTIs [19]. Therefore, monitoring changes in the increase in drug resistance and anticipating these changes seem to be an important medical issue. The mathematical analysis revealed much more complex antibiotic resistance patterns in *E. coli* strains than *P. mirabilis* strains [12]. This finding may suggest that the former are characterized by greater genome plasticity. This work offers a complex analysis of bacterial populations of *E. coli* and *P.* *mirabilis* strains responsible for UTIs, including antibiotic resistance patterns, a mathematical analysis of those patterns, multiplex PCR for the detection of virulence factors, and the correlation of drug resistance patterns with virulence factors. Such a complex approach may allow us to trace the evolution of changes in the most important UTI pathogens, *E. coli* and *P. mirabilis*. The prediction of the emergence of future strain resistance is a prerequisite for the rational planning of medical treatment.

## Abbreviations

UTI: Urinary tract infection
UPEC: Uropathogenic *E. coli*
IPEC: Intestinal pathogenic *E. coli*
ExPEC: Extraintestinal pathogenic *E. coli*
MDR: Multi-drug resistant
